# How to become a good surgeon

**DOI:** 10.1016/j.aopr.2023.01.001

**Published:** 2023-01-12

**Authors:** Peter Wiedemann

**Affiliations:** Leipzig University Medical Faculty, Liebigstrasse 27, 04103, Leipzig, Germany

## 1 Introduction

Good surgeons are essential for patients. The personal prerequisites, the best way to learn and to teach, and the structural requirements and evaluation criteria for the formation of surgeons are described. A definition of a master surgeon is presented. Even if surgical techniques change in the future, the fundamental relationship between surgeon and patient will remain unchanged.

A competent surgeon is one who can "successfully apply professional knowledge, skills, and attitudes"^1^ Almost all residents want to get into surgery as quickly as possible. A surgeon's traditional training seems old-fashioned in a period of artiﬁcial intelligence and robotics with the prospect of replacing humans in some surgical fields. Here I will share some thoughts about the requirements and training of ophthalmic surgeons. Some aspects may be valid for other surgical ﬁelds.

During my residency in ophthalmology at the Cologne University Eye Hospital, Germany, the chairs, Hellmut Neubauer^2^ and Klaus Heimann,^3^ taught three principles: "You can teach anyone to operate", "Not everyone will be a master" and "To operate makes stupid". Meaning: Surgery is a craft that can be learned, but mastery of the craft may make it an art. The performance of routine surgery alone does not guarantee medical quality. Ophthalmic surgery is a delicate craft that requires precise execution, as evidenced by common operations of the anterior and posterior segments of the eye, such as suturing the cornea to avoid distortion or peeling the macula with care not to damage the foveal tissue. To be a good surgeon, certain requirements must be met.

## 2 Prerequisites

### 2.1 Physical requirements

Almost everyone can learn to be an eye surgeon. However, some physical conditions will help to make a successful eye surgeon. It is useful to have four healthy extremities to perform ocular microsurgery, as it is done with two hands and two feet at the same time. Being ambidextrous is an ideal characteristic for a surgeon. Robert Machemer (1933-2009), the father of vitrectomy, tested dexterity with match sticks that needed to be picked up single-handed. For right-handed people, a more advanced test was to use chopsticks with the left hand. Manual dexterity should be practiced and developed with both hands. Stereopsis should be normal to avoid depth perception problems, and normal color vision is favorable to correctly perceiving the red reflex during cataract surgery. Physical deﬁciencies may be overcome by unique engagement and adaption as psychomotor skills can be developed.^4^

### 2.2 Mental requirements

To learn a craft, manual dexterity and good eyesight are important. However, mental requirements are equally or even more critical, talent alone is not enough. As Theodor Fontane said, "Gifts, who would not have them? Talents - toys for children. Only seriousness makes a man. Only diligence makes a genius".^5^ What is more important than dexterity is the mindset. In order to be successful, the student should possess certain traits.

### 2.3 Desire to learn and deliberate practice

In order to improve one's skills, one must focus, study, actively recall, identify gaps, simplify and practice. Having the right mindset is essential, which includes determination, passion, and persistence.^6^ Adult learning involves active, focused learning and getting rid of distractions. The key element is full immersion. This is how many fields, such as surgery, foreign languages, and swimming, can be learned. To become an expert in any field, it is vital to have experience and practice deliberately with a focused goal, slow and attentive perception, and total concentration for many (around 10,000) hours.^7^ Surgical success demands repetition.

### 2.4 Ability to plan

Surgery is a contiguous decision-making process. Planning is essential to achieving a successful outcome. Good surgeons take the time to consider every step before and after surgery. This is a lesson to be learned from the masters of surgery, Hans Goldmann and Hellmut Neubauer, who both advocated for preoperative planning in order to be successful. Hans Goldmann (Director of University Eye Hospital Bern, Switzerland, 1935–1968) writes: By nature, I was not manually skilled: When operating, I, therefore, had to think carefully about every single step and try to understand it rationally.^8^ Hellmut Neubauer (Director of University Eye Hospital Cologne 1966–1986) states: Every operation - except trifles - should be thought through by the surgeon once - with conceivable surprises - beforehand in peace. In rare and predictably tricky situations, I always wrote everything on paper step by step up to the choice of the instrument, often drawing. Then critical spirits from outside could not upset me.^2^ The higher the level of competence, the greater the flexibility one is afforded.

### 2.5 Attention to detail and quality awareness

Surgical failure is often the result of a minor initial mistake, as the consequences may be significant. Therefore, attention to detail is mandatory. Eisner's book "Eye Surgery" explains the intricacies of ophthalmic surgery, including preparation of the surgical field, use of instruments, and the importance of pressure chambers.^8^ Knowing the physical facts is crucial for making the right decisions and getting better results. Following this methodology, a sensible combination of options can help solve surgical problems. My father taught me as a child to be patient and cautious while solving metal puzzles, and the same applies to eye surgery: don't use force. Quality in real time can be seen through good craftsmanship. The tools and skills of the surgeon are important factors, but it is more important to be aware of your abilities and not be deceived. The man with enough insight to admit his limitations comes near perfection.^9^ However, perfection should not be the main goal, but leaving the comfort zone and striving to be better than "good enough" is necessary to achieve quality.

### 2.6 Behavior and respect

Beginners should have basic knowledge of their field, self-criticism, and a little confidence. Preparation is key in ensuring success: one should familiarize themselves with the microscope before going into surgery and sit properly. Young surgeons should not practice alone but instead should begin their surgical career with a mentor for guidance and correction. Mentee and mentor need patience and time. They should demonstrate a friendly, courteous, quiet, and team-oriented attitude in the operating room. Respect must always be paid to the patient's eye, as no eye should be harmed.

## 3 Training of a surgeon

### 3.1 Learning surgery: general aspects

To become a good surgeon, it is necessary to understand surgical indications and interventions, gain experience through observation, participation, and research, and learn from the knowledge and mistakes of other surgeons. Therefore, it is useful to assist to gain the necessary prior knowledge. Instead of making your own mistakes, learn from others. Knowledge without action is useless. Progress in surgical practice can be made quickly by having seniors provide mentorship and later by researching and emulating top performers (by video or visit (Eye must travel). One is stuck in the zone of mediocrity when one is complacent with what one has learned without exceeding the comfort level. Continued medical education and self-education are necessary for independent learning.

Evidence-based medicine combines the best research evidence, clinical expertise, and patient values to optimize medical practices. It requires measuring what can be measured and finding ways to measure what can't so that medical skills can be advanced.^10^ Intraoperative checkpoints are recommended for things that cannot be quantified, such as the ideal amount of peripheral vitrectomy. In-depth analyses of surgical processes are now achievable, making it possible to analyze the inter-patient variability of surgical workflows.^11^

The Dunning-Kruger effect is a cognitive bias in which people with low abilities overestimate their abilities while those with high abilities underestimate them.^12^ It can be measured by comparing self-assessment to performance. To ensure that beginners meet a certain standard of performance, a standardized, internationally valid tool such as the ICO-Ophthalmology Surgical Competency Assessment Rubric (ICO-OSCAR) is useful.^13^ Furthermore, experts must be evaluated by their peers to ensure that surgeons are performing satisfactorily, as there will always be differences in skills even if all surgeons are performing adequately. As surgeons continue to learn and refine their skills throughout their professional lives, they should strive to reach the highest point of their knowledge and skills.^14^

### 3.2 Surgical training

Surgical training should be standardized and structured for newcomers, using simulators and rubrics to reach milestones and improve performance.^15^ Becoming a surgeon is a long process that requires commitment and passion. It is important to plan each surgery and start with the simple tasks, proceeding to the difficult ones. Each surgery has a signature part showing how you can do it (e.g., capsulorhexis, hole localization, or peeling).^16^ Recording and watching surgeries, asking for help when in doubt, and practicing extensively are key to success. Surgeons should be familiar with different techniques and use the most appropriate and least invasive form.

### 3.3 Mentee and mentor

The stages of a surgeon's life involve being a mentee learning from an experienced mentor by watching and getting guidance. The mentor discusses and interprets successes and failures. Most challenges a mentee faces don't arise from something challenging to learn but rather from the teacher, making it seem complicated. As a young surgeon, perform the surgeries your mentor allows while remaining cognizant of your limitations and striving to improve.

Preparation and deliberate practice are essential in gaining the confidence to perform adequately. A successful operation involves not only knowledge but also skills that are often acquired subconsciously. Visual control alone is not enough, as gaining experience and expertise requires more than just a description of how to do the operation. Analyzing intuitively acquired information is difficult and requires feedback from a mentor who can distinguish between conscious and unconscious (in-)competence. A mentor should be respected to achieve the best results when learning to be a surgeon. You get more motivated when you work with someone you admire.

Mentor-apprentice relationships are challenged by the shift to outpatient surgery, which reduces the opportunities to learn from mistakes and refine skills. To be "good" at surgery, one must strive for perfection while being content with doing their best. "Good" is opposed by both "perfect" and "good enough". Perseverance and resilience are key to getting better at surgery in the long run, as time and effort are required to do so.

### 3.4 Books, videos, simulators

A comprehensive book can provide a framework for continuous self-learning. It should provide clear information supported by photographs and videos and emphasize critical decision points. A well-edited book's argumentative strength and well-organized information lead to more clarity and a better-integrated view and understanding. Videos allow sharing of many technical tricks and complications. Simulated surgical training tools can be used to practice skills, but there is no evidence that virtual reality training is better than conventional methods.^17^

## 4 What makes a good surgeon

### 4.1 Characteristics of a good surgeon

The expert surgeon is distinguished by their skills, judgment, experience, and craftsmanship. Their work identifies them. Real-time results are an outstanding feature of ophthalmic surgery. Even if mastery is hard to describe, others - patients and peers - can see when you're doing good work. Mastery involves regularly performing an activity - experts can recognize intricate patterns of information and apply them with ease and flexibility. Mastery is characterized by simplicity in instruments and techniques.^18^ However, even when instruments and technical equipment are absent, a great surgeon can bring out good results.^19^

Mastery is a path, not a destination. It requires instruction, practice, dedication, intentionality, and excellence. Poor lifestyle, ineffective learning and teaching, lack of ambition, laziness, inconsistency, and vanity impede perfection. This journey will never end, and it's impossible to forgo any part of it. We'll never get to the point where we can no longer learn from others. Those who are no longer striving to become better will not remain good.

Reputations are brittle and challenging to uphold. There is a belief that the value of surgical services is determined by the name of that service rather than by the surgeon's skill or the matter to the patient. Such a system would be logical only if all the physicians were equally competent. But all surgeons (and most people who are not) know this is as wrong as believing that all cooks are similarly qualified. Good surgeons can provide a better outcome for the patient than poorer surgeons.

Some surgeons are very vocal about their abilities. John Taylor (1708–1772) was a skilled surgeon who operated on J.S Bach (1685–1750) and had "Qui dat videre dat vivere" (He who gives to see gives life) inscribed on his chariot as self-promotion. Despite his expertise, he operated with the philosophy of "Populus vult decipi" (The world wants to be cheated).^20^ A skilled and ethical surgeon must deliver instead of selling or becoming complacent. I keep with this attitude of Friedrich Philipp Ritterich (1782–1866), the first director of the Leipzig Eye Hospital. I have already demonstrated my expertise in a variety of successful operations and have been supported by many highly qualified colleagues, which is why I do not need to list any more successful cases to be known. It is of no benefit to medical science to report successful cases that follow accepted rules, as this does not provide any new knowledge. What is of scientific value are discoveries made on either known or newly taken paths, but it will be of little value if the reporter is not completely truthful. Therefore, it is essential to report all facts correctly and not leave out any circumstances to support a preconceived opinion.^21^

### 4.2 Patient orientation

As a doctor, always remember that the patient is your only client. Make sure they receive excellent care with equity and integrity and a positive outcome. Don't focus on the image (OCT), examination, or disease, but on how it affects the patient.^22^, ^23^ Put the patient's needs first, and don't be tempted to strive for perfection over care.

Ensure that patients understand the goals and results of surgery. The patient should request an operation from you after your consultation, not the other way around. Inform them that sometimes their issue may require multiple surgeries. Complete each task to the best of your ability. The initial procedure has the highest chance of solving the issue, and the likelihood of success decreases with every additional surgery. A good beginning speaks to a good end (R. Wiedemann).

One should try to perform the procedure in a way one would like to receive. After having surgery on oneself, one may change perspective and even better understand good surgery. One can then be referred to as an "experience expert", someone knowledgeable not because of having studied surgery but because of having experienced it.

### 4.3 Setting the indication

Preoperative planning, postoperative management, and patient discussion are key to surgical success. Setting the indication for surgery is a crucial and increasingly important decision for a physician. A shift towards an economic approach in medical practice has resulted in a subtle re-polarisation of physicians. It takes talent to recognize when to operate on a patient but delaying surgery can sometimes be the best option. The maxim "a cataract is not operated on if it troubles the ophthalmologist, but if it bothers the patient" (Thomas Neuhann) is now more relevant than ever.

### 4.4 Avoiding and handling complications

Complications should be avoided. We distinguish between preventable, complex, and intelligent failure. Simple errors, such as sending the wrong patient to the OR, are preventable failures; complex failure occurs when a procedure is convoluted (vital emergency in the operating room); and intelligent failure is exploratory, such as the development of vitrectomy by Machemer. Checklists help to prevent mistakes.^24^ Surgeons must take care that their team is educated well. Nobody works alone, and good education and preparation prevent many complications. By following these strategies, surgeons can minimize the risk of complications.

A good surgeon needs to be able to deal with complications effectively without creating new ones. The way the complication is handled is more important than the complication itself. It is important to remain calm and consider all options when faced with an unexpected challenge rather than rush into a decision. Taking a moment to breathe and think can make a big difference.

Failure is not the end but a lesson to use going forward. There is always a solution. Use mistakes and failures as data to find better solutions.^25^ In essence, handling complications involves learning what we can influence, what we can control, and what we cannot.

### 4.5 Quality and quantity of surgical interventions

Quality does not usually come before quantity in surgical success (visual success and lack of complications). It depends mainly on a surgeon's competence, judgment, experience, and the number of cases. Decisions for one-time surgery or multiple interventions, knowledge of various surgical options, and ability and flexibility to adapt to the situation are all key factors for successful results.

High-volume cataract surgery leads to improved visual acuity outcomes and decreased complication rates, making it a valuable practice in developing countries with a backlog of cataract cases and a high patient-to-surgeon ratio.^26^ While a larger number of surgeries does not result in further improvement of visual acuity, a surgeon's experience and skill can be developed through years of deliberate, sharp practice. Short-term operating room numbers make a surgeon sharp, deliberate practice over the years makes a surgeon skilled, and the overall volume of a career makes him wise.

### 4.6 Innovation

Inquisitive surgeons who are curious and ask questions are essential to the advancement of the field of medicine. Progress is achieved when unconventional methods are considered (Robert Machemer, 1933–2009). Quickly, what was once astonishing becomes ordinary. Innovation involves developing something new and combining, reexamining, and reviving what has been forgotten.^27^ Playing and working can be complementary, with play helping to develop creativity. To much old information can lead to less openness to learning new things, so it's important to remain mentally agile and open-minded to new ideas. Therefore interests and hobbies outside surgery are useful.

### 4.7 Surgical learning as a function of the medical education system

Ophthalmology separated from general surgery due to the success of ophthalmologists in performing surgery. This and the overall progress of knowledge led to subspecialization. Today, ophthalmic surgery has multiple subspecialties, but it is possible to learn more than one surgical field in ophthalmology once one has had a good surgical introduction.

Surgeons work in a health system that influences their decisions and spectrum of possibilities. The initial start of surgical learning is the most difficult phase. Beginners in countries lacking formal surgical fellowships face added challenges. A desire to learn, combined with goals and a plan, is essential for learning surgery, along with mentorship and self-education. You need resources and a plan, and successful execution. Passion, enthusiasm, and energy play a big role, too.^28^

## 5 Conclusions

Understanding and treating surgical conditions will benefit surgeons, their patients, and new generations of ophthalmologists, even as robots take over certain interventions.

The right mindset and skills are necessary to become a competent surgeon. An excellent surgical mentee needs **P**hysique for extended operations and work night hours, **P**assion for learning and helping patients, **P**erseverance, **P**atience, and **P**syche to endure failures, **P**articipation in being a valuable part of the surgical team, **P**lanning and **P**ortioning of surgical procedures, **P**ractice and **P**recision until **P**erfection is close. Sometimes you need a **P**rayer (S. Natarajan, personal communication).

A master surgeon possesses expertise, is observant of outcomes, is innovative in treatment, can identify complex clinical problems, asks important questions to improve care, disseminates knowledge and expertise, and trains future surgeons.^29^

**Figure:**
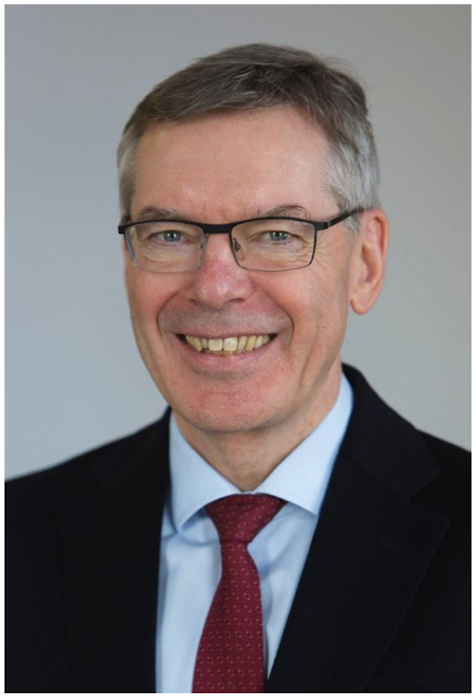

## Study approval

Not Applicable.

## Funding

This research did not receive any specific grant from funding agencies in the public, commercial, or not-for-profit sectors.

## Declaration of competing interest

The authors declare that they have no known competing financial interests or personal relationships that could have appeared to influence the work reported in this paper.
